# Fatigue Severity, Cognitive Strain, and Psychological Health in Long COVID: Untangling the Interconnected Aftermath from a Dedicated Long COVID Clinic

**DOI:** 10.3390/v17121551

**Published:** 2025-11-27

**Authors:** Ashish Bhargava, Hemang Patel, Susan Szpunar, Mamta Sharma, Michael Somero, Shyam Moudgil, Louis Saravolatz

**Affiliations:** 1 Division of Infectious Disease, Department of Internal Medicine, Henry Ford St. John Hospital, Detroit, MI 48236, USA; 2 Thomas Mackey Center for Infectious Disease Research, Henry Ford St. John Hospital, 19251 Mack Avenue, Suite 575, Grosse Pointe Woods, Detroit, MI 48236, USA; 3 Division of Infectious Disease, Department of Internal Medicine, Wayne State University, Detroit, MI 48201, USA; 4 Department of Biomedical Investigations and Research, Henry Ford St. John Hospital, Detroit, MI 48236, USA; 5 Division of Neurology, Department of Internal Medicine, Henry Ford St. John Hospital, Detroit, MI 48236, USA

**Keywords:** long COVID, PASC, fatigue, cognitive impairment, depression, neuropsychiatric symptoms, psychological health

## Abstract

Post-acute sequelae of SARS-CoV-2 infection (PASC) frequently includes persistent fatigue and cognitive dysfunction, but the relationship between these symptoms remains poorly defined. In this prospective observational study at the Henry Ford St. John Long COVID Clinic (LCC) from July 2023 to March 2025, we assessed fatigue severity using the Fatigue Assessment Scale (FAS) and examined its relationship with depression and cognitive symptoms. New patients completed demographic and clinical questionnaires, Patient Health Questionnaire (PHQ)-9, and Montreal Cognitive Assessment (MoCA) at their first LCC visit. Among 41 patients, 35 (85.4%) met the inclusion criteria for fatigue (FAS ≥ 22), with 18 (51.5%) experiencing severe fatigue (FAS > 34). Severe fatigue was significantly associated with shortness of breath, chest pain, and depression. Patients experiencing severe fatigue had significantly higher median PHQ-9 scores (12.5) compared to those with mild to moderate fatigue (5.0, *p* < 0.001). However, there were no significant differences in MoCA scores between these groups. Our study suggests a strong relationship between fatigue and depression in patients with PASC, emphasizing the importance of integrated physical and psychological healthcare. Moreover, since cognitive performance does not vary with fatigue levels, all PASC patients with cognitive dysfunction should receive routine cognitive screenings, regardless of the severity of their fatigue.

## 1. Background

Post-acute sequelae of SARS-CoV-2 infection (PASC), commonly referred to as long COVID, is a growing global health concern that affects millions of people worldwide. It is estimated that between 10% and 30% of individuals experience persistent symptoms beyond the acute phase of COVID-19, regardless of the severity of their initial illness [1,2]. Among these symptoms, fatigue and cognitive dysfunction (“brain fog”) are consistently among the most prevalent, burdensome, and debilitating manifestations [3,4,5,6].

Fatigue is reported by 30–65% of patients with long COVID, often described as severe, persistent, and disproportionate to exertion [4,7]. Cognitive impairment, including deficits in attention, working memory, processing speed, and executive function, affects 20–40% patients months after infection [8,9]. Importantly, these symptoms frequently co-occur; more than half of patients with fatigue also experience concurrent cognitive dysfunction [10]. This clustering has profound consequences: surveys indicate that up to one in four individuals with long COVID are unable to return to work, with fatigue and cognitive impairment cited as the primary reasons [3]. Thus, these symptoms are not only markers of morbidity but also key drivers of long-term disability, healthcare utilization, and societal economic loss.

The clustering of fatigue and cognitive impairment in long COVID parallels observations from other post-viral conditions such as myalgic encephalomyelitis/chronic fatigue syndrome (ME/CFS) and post-Epstein–Barr virus (EBV) illness [11]. Despite this, the relationship between fatigue and cognitive dysfunction in long COVID is still poorly defined. It remains unclear whether these issues are overlapping processes, distinct consequences, or mutually reinforcing impairments. This ambiguity limits efforts to identify at-risk patients and develop effective rehabilitation strategies [3,4,8].

National and international initiatives—including the National Institutes of Health (NIH) RECOVER (Researching COVID to Enhance Recovery) study, the UK PHOSP-COVID cohort (Post-Hospitalization COVID-19 Study), and the World Health Organization’s (WHO) long COVID framework—have recognized fatigue and cognitive dysfunction as core outcomes [12,13,14,15,16]. Despite extensive characterization of fatigue and cognitive symptoms, the interplay between these domains and their psychological correlates remains poorly understood. Systematically evaluating their co-occurrence and impact is therefore essential to inform patient-centered care and address a critical evidence gap [12,13,15].

The current study characterized the clinical characteristics of patients presenting with fatigue symptoms and evaluated their relationship with symptoms of depression and cognitive dysfunction in individuals with long COVID.

## 2. Methods

### 2.1. Study Design and Study Participants

This was a prospective observational study conducted at the dedicated Long COVID Clinic (LCC) affiliated with Henry Ford St. John Hospital, Detroit, MI, between 1 July 2023, and 30 March 2025. Patients were primarily referred to the LCC by primary care or specialty physicians within the Henry Ford Health System following laboratory-confirmed SARS-CoV-2 infection and persistence of symptoms beyond four weeks. All cases were verified by review of electronic medical records before enrollment, ensuring a clinically confirmed rather than self-diagnosed cohort. All participants provided written informed consent prior to study participation. The study was reviewed and approved by the Ascension St. John Hospital Institutional Review Board (Study number—1840011) and the Henry Ford Institutional Review Board. (Study number—17839).

The primary aim of the study was to assess the degree of fatigue among patients with long COVID, using the Fatigue Assessment Scale (FAS), and to evaluate its association with symptoms of depression and cognitive dysfunction over time. Adults ages 18 years and older were included in the initial evaluation at the LCC if they had a confirmed COVID-19 infection (as indicated by a positive PCR or antigen test) and were experiencing symptoms that persisted for four weeks or more after their acute illness. Patients unable to complete a standardized form because of language or literacy barriers, as well as those with pre-existing neurocognitive disorders, were excluded.

Patients participating in the study were asked to complete a standardized case report form that documented their demographic information, comorbidities, and their physical, mental, and emotional health conditions prior to COVID-19. Additionally, on the case report form, patients recorded their symptoms and the severity of their illness during their acute COVID-19 infection, which was categorized as home-based care, hospitalization, or intensive care. They also recorded their symptoms when presenting with long COVID. Fatigue was assessed using the validated FAS, a 10-item self-report questionnaire that measures both physical and mental fatigue [17,18]. Each item is rated on a 5-point Likert scale, resulting in a total score that ranges from 10 to 50 [19]. Patients were categorized into two groups at the time of presentation based on their FAS scores: mild to moderate fatigue (scores from 22 to 34) and severe fatigue (scores above 34) [19].

After being assigned to their respective groups, all patients were asked to complete two assessments: the PHQ-9, which measures the severity of depression, and the Montreal Cognitive Assessment (MoCA), a screening tool for mild cognitive dysfunction. The MoCA is a practical, sensitive and broadly validated tool for screening mild cognitive impairment. Moreover, MoCA scores have been reported to correlate well with more comprehensive neuropsychological batteries [20]. The MoCA evaluates various cognitive domains, including memory, attention, language, and executive functioning [21,22,23,24]. Three of our investigators were MoCA-certified and testing followed standardized procedures.

All assessments were conducted during post-acute follow-up visits, reflecting ongoing or chronic symptoms rather than co-occurring effects of acute infection. All assessments were administered using paper questionnaires and scored by trained clinical personnel.

### 2.2. Study Variables/Covariables

- Key covariables collected at baseline included demographic characteristics such as age, sex, and race.
- Several health-related factors were considered, including body mass index (BMI), smoking status, a history of alcohol or illicit drug use, and existing chronic conditions, as measured by the Charlson Comorbidity Index [25].
- Socioeconomic factors considered included education level, household size, and income.
- Vaccination status was documented in addition to the strains of SARS-CoV-2, identified by the date of initial COVID-19 infection. These strains were categorized as either Pre-Omicron or Omicron. Any episodes of COVID-19 that occurred before the patient’s first visit to the LCC were also noted, along with any hospitalizations because of their acute COVID illness.
- Additionally, the status of each participant’s physical, mental, and emotional health prior to the COVID-19 pandemic was documented, along with symptoms experienced during the acute COVID-19 infection, at the first visit to the long COVID clinic visit, and during subsequent follow-up appointments.

### 2.3. Statistics

Descriptive statistics were calculated to characterize the study group. Normally distributed continuous variables were described as the mean with standard deviation (SD) or median with range. Categorical variables were described as frequency distributions. Univariable analysis was conducted using Student’s *t*-test, the Mann–Whitney U test and the chi-squared test. All data were analyzed using SPSS v. 31.0 and a *p*-value less than 0.05 was taken to indicate statistical significance.

## 3. Results

Of the 41 patients screened for the study, 35 (85.4%) reported clinically significant fatigue (FAS ≥ 22) that met inclusion criteria; 18 (51.5%) met criteria for severe fatigue (FAS > 34). The median duration of their first clinic visit to LCC from their acute COVID was 641 days (range: 46–1571 days). The mean age (SD) of the study group was 54.1 ± 14.4 years, with the majority being 27 (77.1%) female and 28 (80%) White. Of all the patients, 22 (62.8%) reported having attained a bachelor’s degree or a higher level of education. Regarding employment status, eight patients reported being employed full-time, 10 reported part-time employment, two were self-employed, three were receiving disability benefits, and eight were retired. Annual income levels were reported as less than $50,000 by 14 (40%) patients, $50,000 to <$100,000 by 13 (37.1%) and $100,000 or more by eight (22.9%).

The most reported pre-existing comorbid conditions included hypertension (43%), morbid obesity (40%), pre-COVID anxiety (34.3%), pre-COVID depression (34.3%), and asthma (23%). Twelve patients (34.3%) reported tobacco use, 22 (63%) reported alcohol use, and seven (20%) reported marijuana use.

Regarding COVID-19 vaccination, 30 patients (85.7%) had received at least one dose, while 20 (57%) had received three or more doses. Twelve patients (34.3%) reported two or more prior episodes of COVID-19 before their first LCC visit. Nineteen (54.2%) patients were initially infected during the pre-Omicron SARS-CoV-2 waves, while 16 (45.7%) were during the Omicron wave. (Table 1).

During the acute phase of COVID-19, patients who later reported severe fatigue experienced significantly higher rates of shortness of breath, chest pain, and feelings of low mood or depression compared to those with mild–moderate fatigue (Table 1).

Although patients with severe fatigue received a lower number of any COVID-19 vaccine doses and were less likely to have received three or more doses compared to the mild–moderate group, these differences did not reach statistical significance.

At the time of their first LCC assessment, patients with severe fatigue reported a significantly higher prevalence of self-reported symptoms, including shortness of breath, dry cough, loss of appetite, headache, abdominal pain, and muscle pain. In addition to fatigue, the most commonly reported persistent symptoms at the initial LCC visit included muscle pain (77.1%), muscle weakness (77.1%), palpitations (60%), shortness of breath (60%), and dizziness (57.1%). At their first LCC visit, 17 patients (48.6%) reported feeling down, depressed, or hopeless, while 14 (40%) reported little interest or pleasure in doing things.

The median (range) FAS score for all patients was 35 (22–49). The median (range) PHQ-9 score for all patients was 8 (0–22). The median (range) MOCA score for all patients was 24 (19–30). Based on PHQ-9 scoring, 26 patients (74.3%) met criteria for clinical depression. Furthermore, 18 patients (51.5%) reported cognitive impairment, as assessed by the MoCA. (Table 2).

Among patients with severe fatigue, the median FAS score was 39 (35–45), while those with mild to moderate fatigue had a median score of 28 (22–33). Patients experiencing severe fatigue were more likely to report symptoms of depression, hopelessness, and anhedonia (reduced interest or pleasure in activities). The median (range) PHQ-9 score was 12.5 (0–22) in patients with severe fatigue, compared to 5 (1–20) in those without severe fatigue (*p* < 0.001). However, the overall severity classification of depression did not differ significantly between the two fatigue cohorts.

The median (range) MoCA score was 25 (20–30) for patients experiencing severe fatigue, while those without severe fatigue had a median score of 24 (19–30) with a *p* value 0.73. Cognitive performance, as assessed by the MoCA, showed no significant differences between the groups. Additionally, there was no correlation between MoCA scores and the severity of fatigue.

## 4. Discussion

This study investigates the differences between long COVID patients experiencing mild to moderate fatigue and those suffering from severe fatigue, focusing on variations in clinical symptoms, psychological distress, and cognitive performance. Our findings enhance our understanding of long COVID, emphasizing that fatigue is a significant disabling issue closely linked to depression but not to cognitive dysfunction. A key strength of this study is its foundation in a real-world clinical setting, which captures patient experiences and symptom profiles from individuals seeking care for post-COVID sequelae.

Fatigue has consistently been identified as one of the most common and persistent symptoms following acute COVID-19. Our finding that over half (51.5%) of the patients reported experiencing severe fatigue aligns with results from large cohort studies. A large study with 3762 participants found that 77.7% experienced fatigue six months after acute COVID-19 infection, with nearly one-third reporting severe fatigue [3]. Another study indicated that 52.3% of long COVID patients experienced persistent fatigue, independent of initial COVID-19 severity [26]. In our cohort, severe fatigue was associated with a higher symptom burden during both the acute illness and the first LCC visit. This is consistent with earlier research showing that long-term fatigue often co-occurs with multisystem symptoms, including dyspnea, chest pain, headache, and myalgia [27]. These observed patterns suggest that fatigue due to long COVID often represents a broader multisystem syndrome. This phenomenon may be due to ongoing immune dysregulation, as well as inflammatory and oxidative damage. These factors have been proposed as the biological mechanisms contributing to the fatigue and neuropsychiatric symptoms commonly seen in long COVID [28].

Our study found a close link between severe fatigue and depression, as evidenced by both subjective symptom assessments and standardized PHQ-9 scores. This aligns with previous research demonstrating a high prevalence of depression among patients suffering from fatigue related to long COVID. A prospective cohort analysis reported that 31% of patients have depressive symptoms one month after discharge, with fatigue as a significant predictor [29]. Similarly, a recent meta-analysis estimated the prevalence of depression in long COVID to be about 13% and identified fatigue to be among the symptoms most strongly associated with post-COVID neuropsychiatric burden [30]. In our cohort, 26 participants (74.3%) met the criteria for clinical depression. Those who experienced severe fatigue had significantly higher PHQ-9 scores. While the severity of depression did not vary significantly among the different groups based on fatigue levels, the severe fatigue group did exhibit a higher symptom burden, which aligns with findings from previous studies. Patients who experience fatigue are 3.3 times more likely to fulfill the criteria for a diagnosis of depression [31].

In our study, approximately one-third of participants reported a history of anxiety or depression before COVID-19. Although such conditions can influence coping and symptom perception, growing evidence shows that post-COVID anxiety and depression frequently develop regardless of prior mental health history. Our findings are consistent with recent research that demonstrated psychological symptoms are more common among COVID-19 survivors than in those who did not contract the virus, with pre-existing disorders having only a minor influence on this heightened risk [32,33].

The interplay between fatigue, depression, and cognitive dysfunction in the context of long COVID underscores the intricate relationship between physiological and psychological factors. Chronic immune dysregulation, neuroinflammation, persistent stress, and autonomic imbalance have the capacity to disrupt neural circuits integral to the regulation of motivation, mood, and cognition at a physiological level [34,35]. On a psychological level, persistent fatigue can reduce activity levels, increase social isolation, and reduce self-efficacy, which may worsen depression symptoms. These physiological stress responses, together with the psychological burdens of chronic illness, can interact and amplify one another, leading to persistent fatigue. In healthy individuals, fatigue serves as a protective signal encouraging rest and recovery. However, in post-viral conditions such as long COVID, dysfunctional immune and neuroendocrine responses can disrupt this feedback loop, resulting in persistent pathological fatigue [36]. In our study, cognitive impairment was not significantly associated with the severity of fatigue, despite affecting more than half of the participants. This contrasts with a prior study of 100 non-hospitalized patients with long COVID, where 81% experienced cognitive dysfunction and 85% reported fatigue, with both symptoms frequently occurring together [8]. Our findings align more closely with a recent large-scale study in the United Kingdom (UK), which showed that objective cognitive deficits in long COVID have only a weak correlation with self-reported fatigue. This observation suggests that these symptoms could arise from different underlying mechanisms [37]. The lack of correlation between MoCA scores and FAS in our study further supports this hypothesis. The absence of significant group differences in MoCA scores is unlikely to reflect test administration issues, as all assessments were conducted by MoCA-certified investigators following standardized procedures. The MoCA has shown strong criterion and convergent validity with comprehensive neuropsychological batteries, supporting its suitability for this study [38]. Nonetheless, fatigue-related factors—such as reduced engagement or processing speed—could have transiently influenced test performance. Neuroimaging studies have indicated a decrease in gray matter volume in limbic regions along with cognitive deficits after COVID-19 infection [39]. However, it is still unclear if these changes are directly related to fatigue. Together, these findings suggest that fatigue, depression, and cognitive impairment may overlap as symptoms of long COVID, yet each may also have its own distinct pathophysiological profile.

While our study showed a non-significant trend toward lower vaccine uptake among individuals with severe fatigue, evidence from larger studies provides mixed insights. Several investigations have demonstrated that vaccination before or after infection reduces the risk or severity of long COVID symptoms, though the magnitude of protection varies across studies [40,41,42,43]. Overall, these findings suggest that vaccination may mitigate, but not fully prevent, long-term sequelae, such as severe fatigue. However, due to the relatively small sample size, we cannot draw definitive conclusions regarding vaccine-related protection in this cohort.

Our study contributes to a better understanding of symptom patterns in long COVID by comparing the severity of fatigue with mental health and cognitive outcomes. Its strengths include the prospective design, detailed clinical phenotyping and the use of standardized and validated tools (FAS, PHQ-9, MoCA). This study has several limitations, including a small sample size that restricts causal inference and reflects its pilot nature. Relying on self-reported symptoms can lead to recall bias, especially when assessing acute-phase illness retrospectively. We did not include healthy control groups or those with only acute COVID-19 symptoms. Individuals without long-lasting post-acute symptoms or those evaluated during the acute phase may have different diagnostic certainty and clinical evaluation pathways. This approach alleviates misclassification concerns but limits our ability to determine if the link between fatigue and depression is unique to long COVID or part of broader post-infectious phenomena.

Our findings underscore the importance of routine mental health screening in patients with long COVID, particularly those with severe fatigue. Given the high prevalence of depression in this group, integrated physical and psychological rehabilitation programs may improve functional recovery. Moreover, the absence of significant cognitive differences among fatigue groups suggests that all long COVID patients with cognitive dysfunction should receive routine cognitive screenings, regardless of the severity of their fatigue. Future studies with larger, longitudinal cohorts are needed to clarify the trajectory and interrelation of fatigue, depression, and cognitive changes, as well as the impact of COVID-19 vaccination and viral variants on the long-term outcomes.

## Figures and Tables

**Table 1:** Health and Acute Symptom Burden Stratified by Fatigue Level (Data from Initial Long COVID Clinic Assessment). Abbreviations: SD—Standard Deviation; HS—High School; GED—General Educational Development; LCCV—Long COVID Clinic Visit. * *p*-values are from Student’s *t*-test for differences between means, the Mann–Whitney U test where medians are presented and the chi-squared test for associations between categorical variables.

Characteristics	Mild–Moderate Fatigue n (%) n = 17	Severe Fatigue n (%) n = 18	*p*-Value *
**Demographics**			
Age (Mean + SD)	58.1 ±14.9	50.4 ± 13.1	0.11
Sex			0.37
Male	5 (29.4)	3 (16.7)	
Female	12 (70.6)	15 (83.3)	
Race			
White	15 (88.2)	13 (72.2)	
Black	0	3 (16.7)	
Other	2 (11.8)	2 (11.1)	
Body Mass Index (kg/m^2^)	27.9 ± 6.6	32.2 ± 11.1	0.18
**Socioeconomic**			
Education			0.62
HS degree or GED	4 (23.5)	2 (11.1)	
Some Colleges	2 (11.8)	5 (27.8)	
Bachelor’s degree	4 (23.5)	6 (33.3)	
Associate degree	3 (17.6)	2 (11.1)	
Post-graduate	4 (23.5)	3 (16.7)	
Household size			0.41
Less than four persons	15 (88.2)	14 (77.8)	
Four or more persons	2 (11.8))	4 (22.2)	
Employment			- -
Non-employed and disabled	0 (0.0)	7 (38.9)	
Retired	6 (35.3)	2 (11.1)	
Employed at least part time	11 (64.7)	9 (50.0)	
Annual income			0.68
$<25,000	4 (23.5)	5 (27.8)	
$25,000–$49,999	2 (11.8)	3 (16.7)	
$50,000–$99,999	8 (47.1)	5 (27.8)	
$>100,000	3 (17.6)	5 (27.8)	
**Comorbid Conditions at the time of Acute COVID**			
Hypertension	8 (47.1)	7 (38.9)	0.63
Asthma	4 (23.5)	4 (22.2)	0.93
Connective tissue disorder	1 (5.9)	2 (11.1)	0.58
Morbid Obesity	5 (29.4)	9 (50)	0.21
Diabetes	2 (11.8)	1 (5.6)	0.51
Anxiety disorder prior to COVID-19	4 (23.5)	8 (44.4)	0.19
Depression disorder prior to COVID-19	3 (17.6)	9 (50)	0.04
**Charlson Comorbidity Index**: Median (range)	0.00 (0, 1)	0.00 (0, 2)	0.17
**Baseline limitation prior to Acute COVID**			
Sleep-related problem	7 (41.2)	8 (44.4)	0.85
Difficulty remembering	1 (5.9)	3 (16.7)	0.32
Difficulty concentrating	1 (5.9)	3 (16.7)	0.32
Physical disability	2 (11.8)	1 (5.6)	0.51
Mental disability	1 (5.6)	3 (16.7)	0.32
**Any Vaccination dose prior to Acute COVID**	16 (94.1)	14 (77.8)	0.17
**Vaccination doses prior to Acute COVID**			0.8
1–2 doses	5 (31.2)	5 (35.7)	
3 or more	11 (68.8)	9 (64.3)	
**Predicted SARS-CoV-2 Strain Based on the Timing of First reported COVID-19 Infection**			0.6
Non-Omicron	10 (58.8)	9 (50)	
Omicron	7 (41.2)	9 (50)	
**Episodes of COVID prior to initial LCCV**			0.84
One	11 (64.7)	12 (66.7)	
Two	3 (17.6)	4 (22.2)	
Three or more	3 (17.6)	2 (11.1)	
**Symptoms at the time of Acute COVID-19**			
Fever	14 (82.4)	12 (66.7)	0.29
Dry Cough	10 (58.8)	15 (83.3)	0.11
Cough with mucous	10 (58.8)	12 (66.7)	0.63
Shortness of breath	7 (41.2)	15 (83.3)	0.01
Chest tightness	6 (35.3)	9(50)	0.38
Chest pain	4 (23.5)	10 (55.6)	0.05
Running nose	11 (68.8)	15 (83.3)	0.32
Sore throat	10 (58.8)	14 (77.8)	0.23
Headache	14 (82.4)	17 (94.4)	0.26
Dizziness	8 (47.1)	12 (66.7)	0.24
Muscle weakness	10 (58.8)	15 (83.3)	0.11
Muscle pain	11 (64.7)	16 (88.9)	0.09
Diarrhea	3 (17.6)	7 (38.9)	0.16
Abdominal pain	2 (11.8)	4 (22.2)	0.41
Nausea	6 (35.3)	8 (44.4)	0.58
Loss of appetite	8 (47.1)	13 (17.2)	0.13
Rash	3 (17.6)	4 (22.2)	0.74
Tingling and numbness of Hands	7 (41.2)	10 (55.6)	0.4
Tingling and numbness of Feet	5 (29.4)	9 (50)	0.21
Feeling of heart racing	6 (35.3)	7 (38.9)	0.83
Feeling down, depressed or hopeless	4 (23.5)	11 (61.1)	0.03
Feeling little interest in doing things	7 (41.2)	10 (55.6)	0.4

**Table 2:** Prevalence of Reported Symptoms and Psychological/Cognitive outcomes Stratified by Fatigue Level at Initial Long COVID Clinic Visit. Abbreviations: PHQ-9: Patient Health Questionnaire-9.

Characteristics	Mild–Moderate Fatigue n (%)	Severe Fatigue n (%)	*p*-Value
Dry cough	2 (12.5)	9 (50)	0.02
Cough with mucous	3 (18.8)	6 (33.3)	0.34
Shortness of breath	7 (43.8)	14 (77.8)	0.04
Chest tightness	5 (31.3)	8 (44.4)	0.43
Chest pain	4 (23.5)	10 (55.6)	0.05
Running nose	4 (25)	7 (38.9)	0.39
Sore throat	1 (6.3)	3 (16.7)	0.35
Headache	4 (25)	12 (66.7)	0.015
Dizziness	7 (43.8)	13 (72.8)	0.09
Muscle weakness	9 (56.3)	18 (100)	
Muscle pain	10 (62.5)	17 (94.4)	0.02
Diarrhea	1 (6.3)	5 (27.8)	0.1
Abdominal pain	1 (6.3)	8 (44.4)	0.01
Nausea	3 (18.8)	6 (33.3)	0.34
Loss of appetite	1 (6.3)	7 (38.9)	0.025
Rash	1 (6.3)	3 (16.7)	0.35
Tingling and numbness of Hands	6 (37.5)	12 (66.7)	0.09
Tingling and numbness of Feet	5 (31.3)	11 (61.1)	0.08
Feeling of heart racing	9 (56.3)	12 (66.7)	0.53
Feeling down, depressed or hopeless	5 (31.3)	12 (66.7)	0.039
Feeling little interest in doing things	3 (18.8)	11(61.1)	0.012
Depression by PHQ-9	9 (52.9)	17 (94.4)	0.005
Depression Severity			0.22
Mild–Moderate	7 (77.8)	9 (52.9)	
Severe	2 (22.2)	8 (47.1)	
PHQ-9 Median (Range)	5 (1–20)	12.5 (0–22)	<0.001
MOCA Median (Range)	24 (19–30)	25 (20–30)	0.73

## Data Availability

The data set was gathered from the study site and is not accessible to the public.
